# Analysis of sleep quality and quantity during a half-season in world-class handball players

**DOI:** 10.5114/biolsport.2025.148578

**Published:** 2025-03-18

**Authors:** Roger Font, Manuel Ortega-Becerra, Víctor Tremps, Antoni Vicente, Ana Merayo, Milos Mallol

**Affiliations:** 1 Research group in Tecnologia Aplicada a l’Alt Rendiment i la Salut (TAARS), Tecnocampus, Department of Health Sciences, Pompeu Fabra University, Mataró, Spain; 2 GRCE Research Group, National Institut of Physical Education of Catalonia (INEFC), Barcelona, Spain; 3 FC Barcelona Innovation Hub, Barcelona, Spain; 4 Faculty of Sport, Physical Performance and Sports Research Center, Pablo de Olavide University, Seville, Spain; 5 Department of Sports Sciences, Ramon Llull University, FPCEE Blanquerna, Barcelona, Spain; 6 National Institute of Physical Education of Catalonia (INEFC), University of Lleida, Barcelona, Spain; 7 Unidad de Medicina del Deporte, Consejo Catalán del Deporte, Barcelona, Spain

**Keywords:** Recovery, Team sports, Elite team, Heart rate, Sleep, quality

## Abstract

The study aimed to analyse the quality of rest of elite handball players during a half-season and to examine the differences between home and away rest. Adequate rest is key to recovery and performance, but the high density of matches and travel may affect sleep quality and duration. A longitudinal study was conducted over 14 weeks during the 2020–2021 season using sleep monitoring rings in 13 elite handball players to measure physiological and sleep parameters. The variables were compared in different situations such as after a training session, home and away matches, and periods with their national teams. Oura rings were used to collect variables such as heart rate variability, respiratory rate, sleep duration, onset latency and efficiency. Players wore the rings daily excluding training and matches. The data were analysed by comparing the different variables studied. At the physiological level, there were no significant differences between situations. However, moderate differences were found in total time in bed between home matches and travel days (ES = 0.57). There was also less sleep time after matches and travel. Players demonstrated good autonomic flexibility without physiological alterations. However, recovery strategies should be improved as rest time was not adequate after matches and travel. Individual profiles could help detect recovery deficits.

## INTRODUCTION

In high performance sport, it is vital to be able to control and analyse the maximum number of variables involved in the control of the internal and external load in matches and training sessions. This control should help us to improve the performance of our players and minimise the risk of suffering injuries [1].

Nowadays, some technical and medical staff are concerned that focusing only on the training and competition load overlooks how the players respond to this load, without an analysis of the recovery process from the end of a training session or match until the next day when they start the next session [2]. Objective assessments such as countermovement jump analysis [3] or subjective assessments such as the rate of perceived exertion (RPE) [4] or wellness tests to assess fatigue and recovery of athletes [5] are used. It has been shown that the recovery process is key to controlling the load that players have to bear, reducing the risk of injury or a drop in their performance [6]. An excessive accumulation of load or inefficient recovery techniques can lead to an increase in fatigue, whether neuromuscular or cognitive, [7] causing a decrease in the performance of the players or the team [8].

To complement these techniques and develop individualized protocols for each athlete, various technologies are employed to monitor players’ subjective assessments. The main characteristic of these monitoring tools is that they are non-invasive and have a very low cost. One example is the Wellness questionnaires (e.g. Hooper test) [5]. The Hooper test is a reliable and easy to use tool which analyses players’ responses to stress and fatigue by analysing how they cope with the training load, how they recover and how they rest [9]. It has been shown to be valid for assessing the risk of injury, and is a widely used tool [10].

For its part, the RPE seeks to analyse the psychological stress of a training session or competition for the player by looking at the subjective perception of fatigue through the Borg Scale. The RPE is a recognised marker in different investigations where the intensity of exercise and the different homeostatic alterations during physical activity can be assessed [11]. Its use has been validated in handball to obtain the training load [4].

One of the main challenges is the high density of matches per season that players must endure [12]. This intense level of competition, coupled with frequent travel [13], leads to a reduction in both the quality and duration of sleep. As a result, performance in away games may decline, and the risk of injuries can increase [13]. Furthermore, this competitive density can contribute to mood issues and acute or chronic fatigue [13]. Sleep deprivation has been associated with negative changes in mood, such as feelings of frustration, irritability, and decreased confidence, as well as difficulties in emotional regulation [14]. On a cognitive level, sleep restriction has been linked to learning difficulties and impairments in executive functions, which are crucial for the tactical development of athletes during training [15].

Therefore, to safeguard players’ performance and physical health, it is crucial to implement highly efficient recovery processes and rest assessments [13]. These measures are essential to prevent the negative effects of overloading, in terms of both external and internal load [16].

To help the body to speed up these recovery processes and to be able to train or compete in optimal conditions, personalizing the protocols to each team component, different techniques have been developed. These techniques can be of different types: cryotherapy, which has proven to be efficient but expensive and only useful for home matches and training sessions [17]; cold water immersions or temperature contrasts that can be carried out at home or during travel [18]; off-loading massages by medical staff that can be performed anywhere [2]; foam roller [19]; active post-training or post-match recovery [2]. Also, different forms of nutritional support in order to facilitate parasympathetic activation, such as melatonin [20] or curcumin [21], might help to improve the recovery processes.

Assessing sleep quality is crucial for understanding athletes’ recovery processes, as it complements subjective questionnaires with objective measurements [22]. Key elements of sleep quality include duration, intrinsic quality, and circadian timing. The Oura Ring Gen 2 (Oura Health Oy, Oulu, Finland) is a user-friendly device that provides continuous, non-invasive data on sleep patterns, heart rate variability, and body temperature [13]. This technology offers coaches and staff valuable insights into individual recovery needs, allowing for personalized training and recovery strategies. By detecting early signs of overtraining and enhancing athlete engagement through increased awareness of sleep habits, wearable devices like the Oura Ring facilitate a data-driven approach to athlete management. Ultimately, these tools help optimize performance while reducing injury risks, marking a significant advancement in sports science.

The ring provides physiological data on resting heart rate quantification, heart rate variability and respiratory rate via infrared photoplethysmography (PPG), and temperature variation using temperature sensor integrated and used in elite women’s sport [23]. The actigraphy technology, a 3D accelerometer implemented in the ring, provides vital information on the habits of the athletes in terms of sleep time, wake up time, sleep onset latency, sleep efficiency, etc. [23]. It is a non-invasive tool which may provide useful data about recovery and sleep patterns.

After a systematic search, we did not find any research analysing the quality of rest during a season with elite male handball players playing matches at continental level including a number of journeys.

The aim of this research was 1) to analyse the quality of rest in elite handball players and 2) to identify the existing differences between the rest performed depending on the trips, whether sleeping at home or away from home.

## MATERIALS AND METHODS

### Subjects

The study was conducted on 13 world class [24] male handball players from the same team throughout the season (mean ± SD, age: 28.4 ± 3.7 years; height: 188.2 ± 6.1 cm; and body mass: 89.8 ± 12.2 kg). All players were international players with their respective national team’s commitment in the seasonal period in which they participated in this study. The data were obtained from the periodic monitoring of the players during training sessions.

The team competed in the Asobal league (Spanish First Division) and the European Champions League (First Continental competition), winning both at the end of the season.

All subjects had an Oura ring fitted to their middle, ring or index finger, according to their choice. The players gave their consent for the coaches to view the data through the Oura Cloud service. The quality of these data was checked daily by observing the records of all players and determining basic statistical parameters such as the mean and standard deviation from the data recorded in previous days and weeks. The statistics were calculated from downloaded raw data obtained from the Oura server. The data used include the nights without gaps during data monitoring.

### Design

A longitudinal study was conducted to analyse the quality of rest and differences in rest between training, home and away matches and the effect of travel. The monitoring was carried out for 14 weeks (11/03/21 to 16/06/21) during the 2020–2021 season using Oura rings GEN 2 (Oura Health, Oulu, Finland). The rings were used during their daily life, during travel and at night when sleeping. The only time they did not use them was during training and competition, because it was not allowed due to handball rules.

The study was conducted following the ethical principles for biomedical research with human beings, established in the Declaration of Helsinki of the World Medical Association (updated in 2013), and the club’s managerial structure approved its implementation.

All players signed a contractual clause accepting their participation in research projects; therefore approval by an ethics committee was not required [25].

### Data processing

Oura rings (Oura Health, Oulu, Finland) are consumer-based health monitoring devices that measure body signals such as heart rate, respiration rate, body temperature and movement, through infrared photoplethysmography, a negative temperature coefficient and a 3D accelerometer. All sensors are located inside the ring [26]. The device is powered by a USB charging cradle. The model used in this study was Oura Ring Gen 2 (Oura Health Oy, Oulu, Finland).

The main reason for the choice of the Oura device to monitor recovery and sleep variables during different times of the season, always focusing on high level players in field assessment which reproduces as close as possible the handball elite reality, was the level of accuracy provided compared to other portable devices currently on the market [27, 28]. These devices have been validated in the laboratory with an accuracy of 94 to 96% using a sample of 440 nights and 106 participants from various continents. The study analysed the relative impact of different data streams on 2-stage (sleep and wake) and 4-stage classification accuracy (light NREM sleep, deep NREM sleep, REM sleep, and wake) [26].

The variables analysed (Table 1) focused on the root mean square of successive differences between normal heartbeats (RMSSD), defining heart rate variability (HRV), calculated from each successive time difference between heartbeats, with measurements expressed in ms [26, 29, 30]. HRV is an essential variable to understand the parasympathetic branch of the autonomous nervous system, a pillar of the recovery processes, together with the average resting heart rate during the sleep and respiratory frequency (the average number of inhalations per minute) [31]. Respiratory frequency and resting heart rate during sleep are sensitive indicators of physiological state, with fatigue significantly impacting these parameters. When respiratory muscles become fatigued, the body compensates by increasing breathing rate and triggering sympathetic nervous system activity. This adaptation aims to maintain adequate ventilation but can disrupt sleep quality and recovery processes [32]. Sleep parameters were assessed through photoplethysmography and accelerometry to calculate sleep phases and sleep duration. The device calculates total sleep time as minutes spent lying in bed, total sleep time as minutes spent in all sleep phases (light, deep and REM sleep), wake time as minutes spent conscious during the sleep period, sleep efficiency as the ratio of total sleep time to total bed time, and sleep onset latency as the time between lying down and falling asleep. These metrics are derived using a proprietary algorithm, and validation studies indicate an overall agreement of 79% with polysomnography.

**TABLE 1:** Description of the variables analysed

Variable	Definition	Units
RMSSD	The root mean square of successive differences between normal heartbeats	ms
HR Average	Average resting heart rate for the whole night	Lat/min
Breath Average	Average respiratory rate at night	resp/min
Bed total	Total time the player spends in bed	minute
Total Time	Total time the player sleeps that night	Minute
Awake	Total time the player is awake within bedtime	minute
RPE	Players’ subjective perceptions of effort	arbitrary units (au)
GH	Game at home	n
GA	Game away	n
NT	Concentration with the national team	n
R	Rest	n
T	Training Sessions	n
T-T	Training Session + Travel	n

Despite its usefulness, the Oura ring has certain limitations. It may overestimate REM sleep and underestimate deep sleep compared to polysomnography, and its ability to accurately detect periods of wakefulness is limited, with a specificity of approximately 48%. Heart rate variability (HRV) was analysed using the root mean square of successive differences (RMSSD) between heartbeats, a widely used time-domain measure of parasympathetic activity. RMSSD values were averaged across 3-minute intervals during sleep to provide nightly estimates. While RMSSD is a robust and straightforward indicator of vagal tone, it has limitations when used in isolation. It does not fully capture the complexity of autonomic nervous system activity and can be influenced by factors such as age, fitness level, and medication use [26, 29].

The different variables obtained by the ring were analysed according to different situations typical of an elite handball team’s season. A distinction was made between whether the previous day had been a training session (T), a match or a rest day (R). The distinction between home (GH) and away (GA) matches was filtered out [13]. We also analysed the pattern of the players when they were with their national team (NT). Another association that was made was that of training and travel (T-T) to play in another city or country or travel plus rest (typical when playing away from home and returning the same night) [13].

The RPE variable was also used to calculate the fatigue of the players after both matches and training. The players were already familiar with this variable, as it was one of the most common variables used by the staff to monitor the players’ load and fitness. A member of the staff asked the players about this variable within 30 minutes after the end of the training or match. If there was any doubt as to which value the player should answer, the staff member always carried with him the table with the score out of 10 and what each value meant. This evaluation was done on an individual basis so that no other player would know what a teammate was saying [1].

### Statistical analysis

Values are reported as mean ± standard deviation (SD). Statistical significance was established at the level p < 0.05. The distribution of each variable was examined using the Kolmogorov-Smirnov normality test. The statistical significance of differences between variables was tested using the non-parametric Wilcoxon signed-rank test. Effect size (ES) values were calculated using Hedge’s g on the pooled SD [33] with a purpose-built spreadsheet and were interpreted using the thresholds proposed by Hopkins et al. (34) as follows: ES < 0.2, trivial; 0.2 ≤ ES < 0.6, small; 0.6 ≤ ES < 1.2, moderate; 1.2 ≤ ES < 2.0, large; 2.0 ≤ ES < 4.0, very large; and ES ≥ 4.0, almost perfect. The rest of the statistical analyses were performed using SPSS software version 26.0 (IBM Corp., Armonk, NY). Figures were designed using GraphPad Prism version 9.4.1 (GraphPad Software, Boston, MA).

## RESULTS

Table 2 summarizes each variable’s mean value and standard deviation, effect size and statistically significant differences between average resting heart rate and breathing variables according to activity. No significant differences were observed in any of the various scenarios considered, except for the average heart rate (HR average) between the situations in NT (p < 0.005), although the effect size is trivial when comparing the rest of the situations (ES < 0.2; ES < 0.6).

**TABLE 2:** Effect size and statically significant differences between average resting Heart Rate and Breath variables according to activity.

Variables	GH	ES	GA	ES	NT	ES	R	ES	T	ES	T-T
Rmmsd (ms)	88.8 ± 33.6		90.1 ± 35.2		83.5 ± 33.6		85.1 ± 37.1		85.9 ± 33.3		88.6 ± 31.7
		GA:0.03		NT:0.01		R:0.001		T: -0.08		T-T: -0.07	
		NT:0.08		R:0.18		T: -0.07		T-T: -0.16			
		R:0.15		T:0.12		T-T: -0.15					
		T:0.08		T-T:0.04							
		T-T:0.01									

HR Average (lat/min)	48.7 ± 5.7		48.7 ± 7.2		50.6 ± 5.5^T^		50.8 ± 7.8		49.4 ± 6.5		49.1 ± 5.2
		GA: -0.01		NT: -0.29		R: -0.02		T:0.19		T-T: 0.04	
		NT: -0.32		R: -0.27		T: 0.04		T-T:0.22			
		R: -0.28		T: -0.11		T-T: 0.27					
		T:0.11		T-T: -0.07							
		T-T: -0.7									

Breath Average (resp/min)	15.2 ± 2.0		15.2 ± 2.0		15.5 ± 2.0		15.5 ± 2.1		15.2 ± 2.0		15.2 ± 1.9
		GA: 0.02		NT: -0.11		R: -0.02		T:0.17		T-T: -0.03	
		NT: -0.14		R: -0.13		T: 0.15		T-T:0.14			
		R: -0.15		T: 0.04		T-T: 0.13					
		T:0.01		T-T: 0.01							
		T-T: -0.01									

ES: effect size; Rmssd: root mean square of successive differences between normal heartbeats; HR average: Average resting heart rate for the whole night; Breath average: average respiratory rate at night; GH: Game at home; GA: Game away; NT: National Team Activity; R: Rest; T: Training Session; T-T: Training Sessión + Travel. Significant differences (p < 0.05): ^T^ Training Session.

Table 3 shows the effect size and statistically significant differences between rest variables according to activity. In relation to Bed Total, the players show differences between GH and R, and between R and T and R and T-T (p < 0.05), although the effect sizes found were small (GH and R ES = 0.46; R and T ES = 0.34; R and T-T ES = 0.57). Total Time also shows a similar trend among the different scenarios analysed, and the effects sizes found are trivial (ES < 0.2) or small (ES < 0.6), although some significant differences were observed between GH and NT (p < 0.05 ES = 0.47) and R (p < 0.001 ES = 0.58). In addition, differences were also found between R and T (p < 0.05) with a trivial effect size (ES = 0.00).

**TABLE 3:** Effect size and statically significant differences between resting variables according to activity.

Variables	GH	ES	GA	ES	NT	ES	R	ES	T	ES	T-T
Bed Total (minute)	514.7 ± 54.3^R^		499.5 ± 84.8		486.7 ± 78.4		472.7 ± 87.3 T,T−T		493.2 ± 61.4		479.9 ± 65.9
		GA: -0.21		NT: 0.15		R: 0.10		T: -0.21		T-T: 0.20	
		NT: 0.41		R: 0.24		T: -0.09		T-T: -0.02			
		R: 0.46		T: 0.09		T-T: 0.09					
		T: 0.34		T-T: 0.24							
		T-T: 0.57									
Total Time (minute)	440.7 ± 46.1^NT, R^		418.9 ± 71.0		414.0 ± 69.6		398.1 ±80.1^T^		419.5 ± 60.8		413.3 ± 64.2
		GA: -0.36		NT: 0.06		R: 0.20		T: 0.00		T-T: 0.00	
		NT: 0.47		R: 0.25		T: 0.02		T-T: -0.21			
		R: 0.58		T: 0.08		T-T: 0.02					
		T: 0.43		T-T: 0.43							
		T-T: 0.43									
Awake (minute)	74.0 ± 34.9		80.6 ± 37.5		72.6 ± 41.2		74.6 ± 33.3		73.7 ± 34.7		66.6 ± 29.8
		GA: 0.17		NT: 0.19		R: -0.05		T: 0.02		T-T: 0.20	
		NT: 0.03		R: 0.16		T: -0.03		T-T: 0.23			
		R: -0.01		T: 0.18		T-T: 0.15					
		T: 0.01		T-T: 0.38							
		T-T: 0.21									

ES: effect size; Bed Total: Total time the player spends in bed; Total Time: Total time the player sleeps that night; Awake: Total time the player is awake within bedtime; GH: Game at home; GA: Game away; NT: National Team Activity; R: Rest; T: Training Session; T-T: Training Session + Travel. Significant differences (p < 0.05): ^R^ Rest; ^T^ Training Session; ^T−T^ Training Session+Travel; ^NT^ National Team Activity. Significant differences (p < 0.001): ^**R**^ Rest.

**FIGURE 1:**
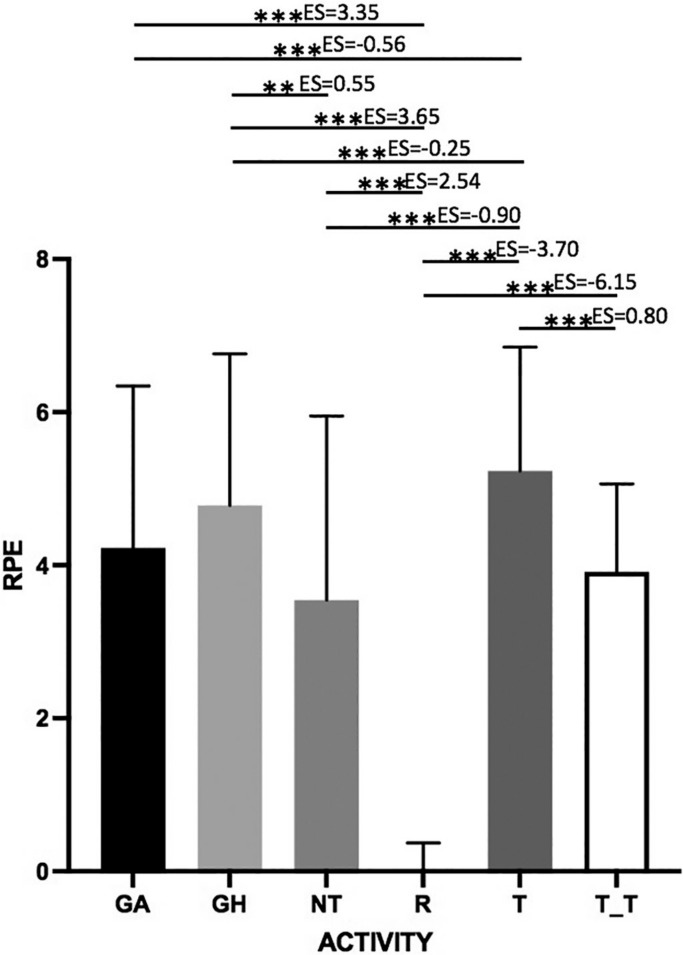
RPE registered by type of activity. GH: game at home; GA: game away; R: rest; T: training session; T-T: training session + travel. The thickness of the lines represents the magnitude of the difference (effect size). < 0.2 trivial; 2.0 < d < 4.0 very large; ≥ 4.0 almost perfect ** p < 0.01; ***p < 0.001 session + travel. Significant differences (p < 0.05): ^R^ rest; ^T^ training session; ^T−T^ training session+travel; ^NT^ national team activity. Significant differences (p < 0.001): ^**R**^ rest

Figure 1 shows graphically the results obtained in relation to the RPE in each of the situations and the differences found in the responses reported by the players. It can be observed how the perception of effort reported by the players in R and T situations indicates the existence of significant differences from the rest of the situations studied (p < 0.001) with very large (ES < 4.0) or almost perfect (ES ≥ 4.0) effect sizes, except between T and T-T, in which the effect size was trivial (ES < 0.2). Another aspect to highlight is that between the competition situations (GH and GA); no significant differences were found.

## DISCUSSION

The aim of this research was to analyse the quality of rest of elite male handball players during a 14-week period within the competitive season, and also to observe the relevant differences between the different rest situations typical of an elite team (T, R, GH, GA, NT, T-T).

The main findings of the research were that no significant differences were found at the physiological level, but significant changes were observed in the total rest time.

On a physiological level, no significant differences were found when comparing the different variables such as heart rate variability, specifically with the variable root mean square of successive differences between normal heartbeats (RMSSD), the HR average and the average respiratory rate with the different rest situations. Other research has found that elite volleyball players, for example, before important play-off matches, also had no significant differences in HR variability [35], corroborating the results of our research.

All these physiological variables studied in the current research are related to the activation of the autonomic nervous system. In elite athletes, the flexibility of the autonomic nervous system (ANS) plays a crucial role in performance and recovery. This flexibility manifests in two key ways: activation through the sympathetic nervous system during stressful situations, such as competitions or intense training sessions, and deactivation via the parasympathetic nervous system to prioritize recovery and the assimilation of training loads. The main objective is to optimize this autonomic balance, enabling athletes to respond effectively to challenges and recover efficiently [36]. Furthermore, elite athletes must maintain this autonomic flexibility across various situations, including sleep after training sessions or travel, resting on different mattresses, and periods spent with national teams. These varied circumstances should not significantly impact their rest quality or autonomic function. In line with these results, D’Ascenzi et al. [35] did not find significant differences at the level of the autonomic nervous system. This aspect reinforces the professional context in which the players involved in the study find themselves, showing good flexibility to change from one system to another and adapt to the stimulus presented to them, if this stimulus does not become a chronic overload.

Previous research revealed that handball players with a non-competitive weekend, increasing passive rest time, showed an improvement of the autonomic nervous system through the control of heart rate variability [37]. Conversely, poor rest might lead to reduced performance and a reduction in heart rate variability [38].

Moreover, the diverse array of travel, training sessions, and match situations does not significantly impact the players’ capacity to activate their sympathetic nervous system for exercise or to transition into a more anabolic phase characteristic of recovery processes, which is primarily mediated by the parasympathetic nervous system (Table 2). This physiological adaptability underscores the robustness of elite athletes’ autonomic nervous system regulation across various environmental and competitive contexts [36].

In contrast to our results, Myllymäki et al. [39] observed physiological alterations in rest after training in non-elite athletes. Other research concluded that the effects of sleep loss on physiological responses to exercise appear to lead to a reduction in the quality and quantity of sleep and may result in an imbalance of the autonomic nervous system, mimicking the symptoms of overtraining syndrome [40].

The predominant travel method involved air transport followed by bus journeys, which significantly delayed the players’ return to their accommodation. Consequently, these extended travel protocols resulted in a substantial postponement of the athletes’ usual sleep onset time (Table 3), thereby reducing their overall rest duration and potentially impacting recovery and performance parameters. Different strategies should be considered to improve the quality of rest on training plus travel days: traveling in better conditions, following better schedules to try to maintain the same rest time as when at home, employing physiological recovery strategies upon arrival at the destination (e.g. aerobic running, massage, mobility training). Staff should consider these options to prevent a reduction of the players’ performance or an increase of the risk of injury [2, 7].

A moderate difference is observed between home match days and rest days, with a reduction in sleep time on recovery days. A possible explanation is that on recovery days, the night analysed is after a competition or trip, which means that players tend to go to sleep later than usual, with greater stimulation of the sympathetic nervous system, showing difficulties falling asleep and relaxing [36]. This quality and duration of sleep have been examined in previous studies [13].

These results are similar with other researchers who found that among football players there was a decrease in the total post-match rest time in both boys [30] and girls [29] during an official tournament. Post-competition rest issues have also been observed in rugby [13]. Moreover, 52.3% of athletes who play team sports showed post-match sleep disorders [13]. Power et al. [41] detected poorer sleep quality after the game and the MD-1 in female basketball players.

The findings of this study contrast with previous research on young elite football players, where no decrement in sleep quality or quantity was observed following high-intensity training or various pre-rest recovery strategies, including cold-water immersion [42]. Furthermore, our results diverge from studies reporting that players slept longer when away from home, attributed to stricter schedules and team concentration [36]. In the present study, time awake in bed decreased, and sleep onset difficulty increased during away games.

Regarding the relationship between RPE and the analysed situations, our findings suggest that effort perception is not significantly influenced by the familiarity of the context (home vs. away games). This contrasts with Fox et al. [43], who found differences in sleep time and perception among basketball players as a function of training load. Similarly, in women’s football, moderate to small negative correlations were observed between RPE and sleep duration and efficiency [29].

The discrepancies in our results may indicate that elite men’s handball players have adapted to the competitive demands involving travel, training, and irregular rest periods without substantial alterations in their perception of effort. This adaptation could be attributed to the specific characteristics of handball and the players’ experience level. However, further research is needed to elucidate the mechanisms underlying this apparent resilience and to investigate potential long-term effects of such adaptations on player health and performance.

These findings underscore the complexity of the relationship between travel, competition, sleep patterns, and perceived exertion in elite sports. They highlight the need for sport-specific and context-sensitive approaches when designing training and recovery strategies for high-level athletes.

## Limitations

The main limitation of this research is that only 14 competitive weeks of the same team were analysed. The players used the rings to control their rest every day during these weeks, including the training camps with their national teams. In addition, as handball is a sport with physical contact, in line with the regulations, the rings could not be used during training and matches due to the risk of injury to the player, teammates or opponents.

## Practical applications

The results of this study highlight the need to improve players’ rest and recovery strategies, despite the lack of significant differences in the measured variables, including RPE. The data suggest that rest time was suboptimal, indicating a need for technical staff to enhance recovery protocols to reduce injury risk. The key aspect to address is the gap between actual and optimal rest time. This can be achieved by focusing on reducing sleep onset latency, minimizing wake time during sleep, and mitigating factors that disrupt sleep quality. By implementing targeted interventions tailored to individual needs and the specific demands of elite handball, technical staff can optimize players‘ recovery, potentially improving performance and reducing injury risk [44, 45].

## CONCLUSIONS

No previous studies that analysed rest variables in an elite men’s handball team during a regular season were found. The main findings of the present study indicated no significant differences in physiological and perceived effort variables across different team sessions. However, differences were observed in rest quality and duration. Therefore, the technical staff of handball teams should implement strategies to enhance the quality of rest and increase players’ sleep duration. These measures aim to optimize recovery from training sessions, matches, and travel-related exertion. Additionally, they may help mitigate injury risk and prevent player overreaching. Such interventions could contribute to improved overall performance and athlete well-being throughout the competitive season.

Future lines of research could investigate which of these different strategies could be useful in elite handball teams to try to ensure that players’ rest is of the same quality when competing at home and away. Nowadays there are many more recovery tools and strategies, but we still observe that there is a decrease in the quality of sleep when teams travel.
